# Odontogenic Fibromyxoma and Odontogenic Cyst in an Eight year old Boy: Three-year Follow-up

**DOI:** 10.5681/joddd.2009.026

**Published:** 2009-09-16

**Authors:** Hossein Shahoon, Mostafa Esmaeili, Maryam Nikhalat, Ensie Farokhi

**Affiliations:** ^1^Assistant Professor and Head, Department of Oral and Maxillofacial Surgery, Faculty of Dentistry, Shahed University of Medical Sciences, Tehran, Iran; ^2^Senior Resident, Department of Oral Medicine, Faculty of Dentistry, Shahed University of Medical Sciences, Tehran, Iran; ^3^Dentist, Private Practice, Tehran, Iran

**Keywords:** Mandible, odontogenic cyst, odontogenic fibromyxoma

## Abstract

Odontogenic fibromyxoma is a rare and locally-invasive benign neoplasm found exclusively in the jaws. It has the potential for extensive bony destruction and extension into the surrounding structures. In the presented case, radiographic and histological features as well as the treatment and follow-up of odontogenic fibromyxoma accompanying odontogenic cyst of mandible in an 8-year-old boy are discussed.

## Introduction

Odontogenic myxoma is a rare and locally-invasive benign neoplasm found exclusively in the jaws. It is a benign odontogenic tumor and should not be confused with soft tissue myxoma, which is a relatively common tumor of the soft tissues. Odontogenic myxoma usually occurs in the tooth-bearing areas. Therefore, it is supposed to be of tooth origin, and derived from the mesenchymal portion of a tooth germ—most likely of the dental papilla.^1^ It has the potential for extensive bony destruction and extension into the surrounding structures. Almost 75% of odontogenic myxomas occur in patients around 23-30 years of age with a slight female predilection. It rarely occurs in patients over 50 or under 10 years of age.^1 - 4^ The tumor occurs almost equally in the maxilla and mandible with a slight predilection for the posterior mandible.^1^ A few cases are described in the ramus, condyle and non-tooth bearing areas.

Radiographically, the majority of cases present as expansile mass and multilocular radiolucency with or without scalloped borders, although some are unilocular, and rare cases present with a diffuse and mottled appearance which can be mistaken for a malignant neoplasm.^1 , 3 - 5^ The more common fibrotic odontogenic myxomas (also known as odontogenic myxofibroma or fibromyxoma) have larger tumor bodies, which makes their curettage easier.

Histologically, odontogenic myxoma is made up of loose and delicate fibrous connective tissue. The fibroblasts are stellate and are suspended on a delicate network of collagen fibrils.^6,7^

Although odontogenic cysts are relatively common in the jaws, co-occurrence of odontogenic myxoma with an odontogenic cyst is a rare finding, as reported in the present case.

## Case Report

An 8-year-old boy was referred to a private clinic of oral and maxillofacial surgery. The patient suffered from a mild swelling (mass) with tenderness on the right side of mandible for a period of six month. The skin over the swelling area was normal and there was no local rise of temperature. On palpation, the mass was immobile with bony hard consistency. Buccal and palatal cortexes were expanded and there was no history of paresthesia or hyposthesia. The lesion extended from mandibular right second premolar to the half height of the mandible ramus (Figure 1).


Figure 1. Extra-oral photograph showing a swelling in the right side of mandible (a). Intra-oral photograph showing an expansion in buccal and lingual cortex of right angle of mandible (b).

**a:**
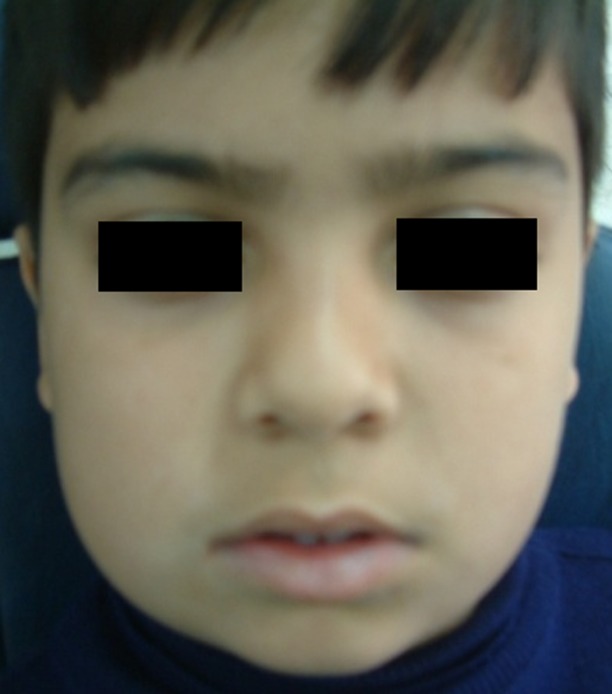

**b:**
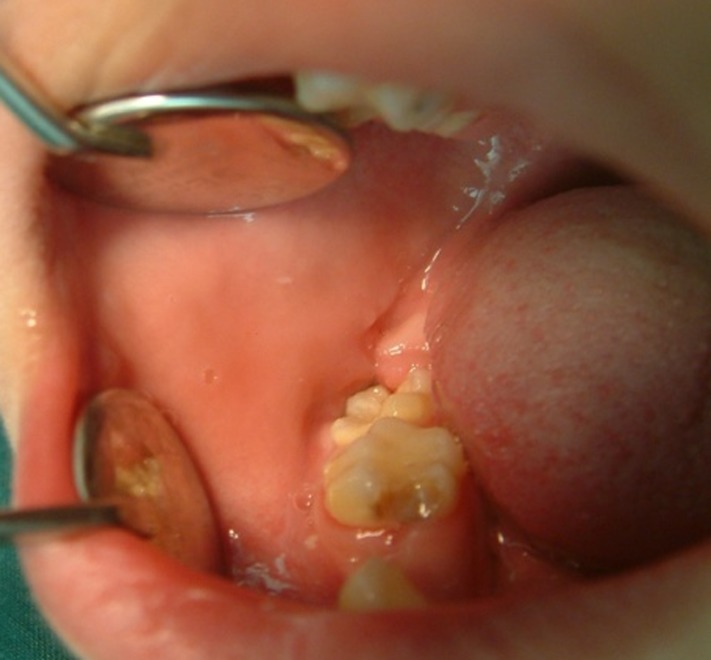

### Radiographic Examination

**Figure 2:**
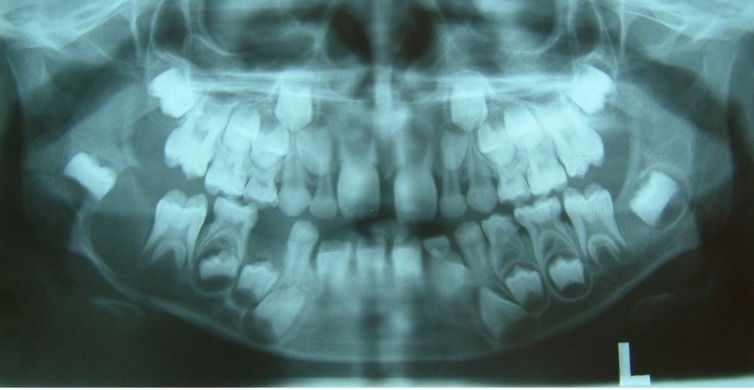
Panoramic radiograph showing a well-defined, unilacullar radiolucency around mandibular right second molar as well as a multilacullar radiolucency extending from distal of mandibular right first premolar to the mesial of mandibular right second molar.

The panoramic radiograph revealed a well-defined, unilacullar radiolucency involving mandibular right second molar and a multilacullar radiolucency on the distal of mandibular right first premolar to the mesial of the right second molar (Figure 2).

### Treatment

The treatment consisted of surgical excision of the lesion under local anesthesia with frozen sections during the surgery sent for pathological examination to reveal whether the lesion is benign or malignant and the margins are free of lesion or not. After several frozen sections, clear bony margins were reached to prevent recurrence of the neoplasm. Mandibular right second molar was also extracted. Pathologic lesion involved a neurovascular bundle (inferior alveolar nerve) that was separated meticulously from the lesion.

### Histopathologic Examination

The biopsy consisted of an amorphous, irregular mass sized 3.5 × 2 × 0.2 cm^3 ^. The biopsy also contained the crown of a molar tooth. Fibromyxomatic tissues consisting of bundle and round cells with blood vessels and collagen fibers in a bright matrix were seen. In some areas of the lesion, structures of odontogenic cyst with inflammatory features were found (Figure 3).


Figure 3. (a) Medium-power image exhibiting cells with blood vessel and collagen fiber in loose matrix (hema-toxylin-eosin, original magnification ×100). (b) High-power image exhibiting round and boundle cells (hematoxylin-eosin, original magnification ×400)

**a:**
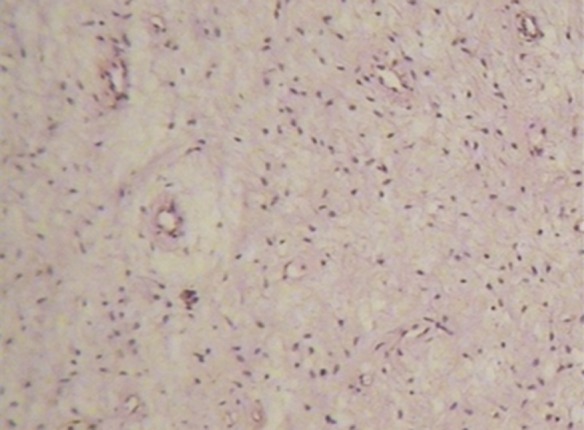

**b:**
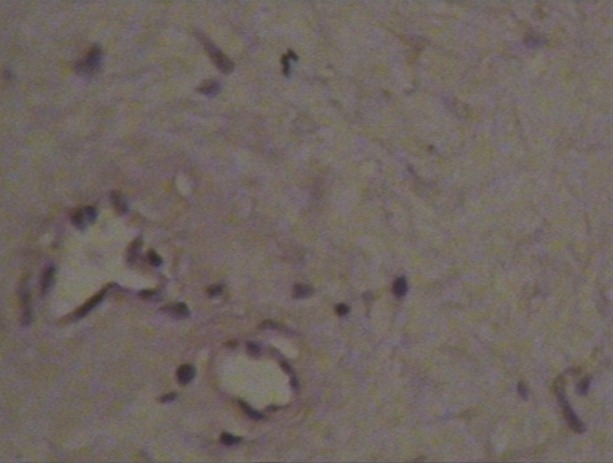

###  Follow-up

**Figure 4:**
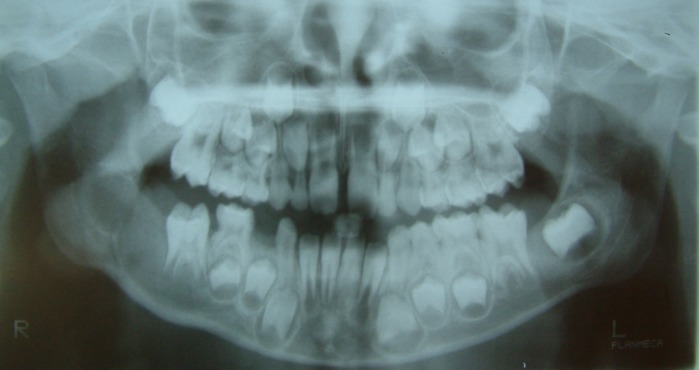
Panoramic radiograph 4 weeks after surgery.

**Figure 5:**
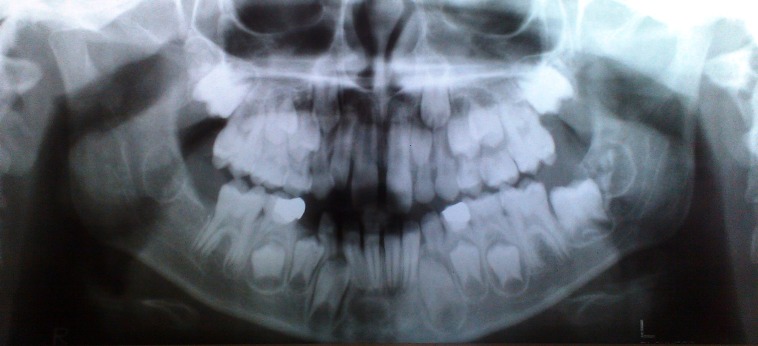
Panoramic radiograph 3 years after surgery.

Immediate post-operative follow-up was scheduled weekly for one month, monthly for the next five months and twice a year for the next three years. In the first week, anesthesia or hypoesthesia was not detected. A panoramic radiograph 4 weeks after surgery showed relative healing of the surgical site (Figure 4). Panoramic Radiograph 3 years after surgery showed no recurrence (Figure 5).

## Discussion

Odontogenic myxoma is a rare and locally invasive benign neoplasm found exclusively in the jaws. It commonly occurs in the second and third decades of life and the mandible is involved more commonly than the maxilla. The lesion often grows without symptoms and presents as a painless swelling.^8^ The radiographic features are variable, and the diagnosis is, therefore, not easy.^8^ According to variable clinical and radiographic appearance, it should be considered in the differential diagnosis of radiolucent lesions of both jaws in all age groups.^9 - 11^ According to the radiographic feature, site, and consistency of lesion in the present case, the differential diagnosis was odontogenic myxoma, mural ameloblastoma and infected odontogenic cyst.^12^ However, according to the pathological findings of the case, final diagnosis was odontgenic fibromyxoma accompanied with odontogenic cyst.

Being rare in children, occurrence of odontogenic fibromyxoma in the first decade and its combination with odontogenic cyst was the most considerable finding of this case.
